# Role of Immune Cell-Specific Hypermethylation Signatures in Classification and Risk Stratification of Breast Cancer

**DOI:** 10.3389/fmed.2021.674338

**Published:** 2021-08-26

**Authors:** Yong Chen, Fada Xia, Bo Jiang, Wenlong Wang, Xinying Li

**Affiliations:** Department of General Surgery, Xiangya Hospital, Central South University, Changsha, China

**Keywords:** immune cells, DNA methylation, tumor immune microenvironment, breast cancer, risk stratification

## Abstract

**Background:** Epigenetic regulation, including DNA methylation, plays a major role in shaping the identity and function of immune cells. Innate and adaptive immune cells recruited into tumor tissues contribute to the formation of the tumor immune microenvironment (TIME), which is closely involved in tumor progression in breast cancer (BC). However, the specific methylation signatures of immune cells have not been thoroughly investigated yet. Additionally, it remains unknown whether immune cells-specific methylation signatures can identify subgroups and stratify the prognosis of BC patients.

**Methods:** DNA methylation profiles of six immune cell types from eight datasets downloaded from the Gene Expression Omnibus were collected to identify immune cell-specific hypermethylation signatures (IC-SHMSs). Univariate and multivariate cox regression analyses were performed using BC data obtained from The Cancer Genome Atlas to identify the prognostic value of these IC-SHMSs. An unsupervised clustering analysis of the IC-SHMSs with prognostic value was performed to categorize BC patients into subgroups. Multiple Cox proportional hazard models were constructed to explore the role of IC-SHMSs and their relationship to clinical characteristics in the risk stratification of BC patients. Integrated discrimination improvement (IDI) was performed to determine whether the improvement of IC-SHMSs on clinical characteristics in risk stratification was statistically significant.

**Results:** A total of 655 IC-SHMSs of six immune cell types were identified. Thirty of them had prognostic value, and 10 showed independent prognostic value. Four subgroups of BC patients, which showed significant heterogeneity in terms of survival prognosis and immune landscape, were identified. The model incorporating nine IC-SHMSs showed similar survival prediction accuracy as the clinical model incorporating age and TNM stage [3-year area under the curve (AUC): 0.793 vs. 0.785; 5-year AUC: 0.735 vs. 0.761]. Adding the IC-SHMSs to the clinical model significantly improved its prediction accuracy in risk stratification (3-year AUC: 0.897; 5-year AUC: 0.856). The results of IDI validated the statistical significance of the improvement (*p* < 0.05).

**Conclusions:** Our study suggests that IC-SHMSs may serve as signatures of classification and risk stratification in BC. Our findings provide new insights into epigenetic signatures, which may help improve subgroup identification, risk stratification, and treatment management.

## Introduction

Breast cancer (BC) is the most common cancer among women worldwide. Despite significant advances in locoregional therapies, endocrine therapies, chemotherapy, and molecular targeted therapy, BC remains the second leading cause of cancer-related deaths among women (1, 2). Immune evasion has recently been recognized as a hallmark of tumor progression. Tumors can induce local immune dysregulation by suppressing innate and adaptive immune responses in BC (3). The tumor immune microenvironment (TIME) is an important part of the tumor microenvironment. It is highly heterogeneous and plays an important role in tumor progression and disease prognosis in various cancers (4). Therefore, accurate classification of disease based on the TIME is crucial for the assessment of prognosis, as well as to aid in making treatment decisions.

The rapid development of high throughput technologies has enabled the identification of the transcriptomic signatures of immune cells. Several tools based on the use of information about the transcriptomic signatures of immune cells have been successfully used for the classification and risk stratification of various cancers, including BC (5–7). These tools include ESTIMATE, TIMER, and CIBERSORTx, which were created to assess the conditions prevailing in TIME, as well as indicators such as immune score and immune cell population. Unlike the transcriptomic signatures of immune cells, methylation signatures are heritable and reversible, and can be adjusted rapidly in the course of an immune response, to appropriately regulate the progression of immunity. However, the methylation signatures of immune cells have not been thoroughly investigated, and it is not clear whether they would be useful for classification and risk stratification in BC.

The primary tumor, regional lymph nodes, distant metastases (TNM) staging system, established by the American Joint Committee on Cancer (AJCC)/Union for International Cancer Control (UICC), is the most commonly used risk stratification system in BC (8). Prognostic information provided by this system, although useful, is incomplete, and many studies have shown that incorporating additional clinicopathological and molecular characteristics, such as tumor differentiation grade, non-coding RNA, mutation status, immune score and microsatellite instability, may improve the accuracy of prediction of prognosis (9–11). Due to the rapid advances that have been made in methylation sequencing technology, single-base resolution has been achieved, and a large number of methylation signatures have been discovered and defined as biomarkers of prognosis in BC (12, 13). Therefore, we speculated that immune-related methylation signatures may be useful for risk stratification.

DNA methylation is a dynamic epigenetic modification, which plays a prominent role in determining cell development and lineage identity, especially in the immune system (14). During the differentiation of hematopoietic stem cells into innate and adaptive immune cells, methylation events, including hypermethylation and hypomethylation, facilitate the commitment of these cells to a lymphoid or myeloid fate, thereby establishing the identities of differentiated cell types (15). Therefore, immune cell-specific methylation signatures may be closely associated with the function of the cells in immunity. Due to the association between methylation events and immune cells, which is compounded by the association between immune cells and tumor progression, specific methylation signatures of immune cells may play an important role in classification and risk stratification of cancers. In this study, we focused on the role of immune cell-specific hypermethylation signatures (IC-SHMSs) in the classification and risk stratification of BC. Specific hypermethylation signatures of six immune cell types were separately identified by analyzing methylome data. Unsupervised hierarchical clustering analysis and Cox proportional hazard models were used to explore the roles of these signatures in the classification and risk stratification of BC patients.

## Materials and Methods

### Data Collection

DNA methylation profiles of immune cells from eight datasets (GSE35069, GSE83156, GSE88824, GSE61195, GSE130030, GSE130029, GSE131989, and GSE87095) based on the Illumina HumanMethylation 450 (450K) platform were downloaded from the Gene Expression Omnibus (GEO) database. Immune cell samples from patients with immune-related diseases were filtered out, leaving only normal samples, and all these immune cell samples were isolated from peripheral blood. The Cancer Genome Atlas (TCGA) level 3 gene expression data normalized by fragments per kilobase of exon per million reads mapped (FPKM), DNA methylation data, and somatic mutation data (mutect2) from BC patients, which were downloaded from the National Cancer Institute's Genomic Data Commons Portal (GDC; https://portal.gdc.cancer.gov/); matched survival and clinical information were obtained from the University of Santa Cruz (UCSC) Xena database (http://xena.ucsc.edu/). Only primary BC samples marked with barcode 01A from TCGA database were retained for our study.

### Identification of the IC-SHMSs

The methylation profiles of immune cells were quality controlled and normalized separately using the R package “ChAMP” and combined after correcting for batch effects between different datasets using the ComBat method associated with the “ChAMP” package (16). Principal component analysis (PCA) was used to test the quality of immune cell samples showing methylation profiles, using the R package “FactoMineR” (17). Using the champ.DMP function in the “ChAMP” package with the cutoff adjusted to *p* < 0.05 and deltabeta >0.2, five differential analyses were conducted between CD8^+^T cells and the other five immune cell types separately. Five sets of significant hypermethylated probes of CD8^+^T were identified, and the intersection of these five sets was defined as the IC-SHMSs of CD8^+^T cells. In a similar manner, the IC-SHMSs of the other five types of immune cells were also identified separately. Moreover, we performed univariate and multivariate Cox regression analyses to identify the prognostic value of these IC-SHMSs, using the survival information and methylation profiles of BC.

### Identification of the Subgroups of BC Patients

To identify subgroups of BC patients, we used the list of IC-SHMSs showing prognostic value to perform unsupervised hierarchical clustering of BC patients using the R package “cluster.” The distances between BC samples were calculated using the Euclidean method, and clustering was accomplished *via* the ward.D2 method. In addition, we utilized the Calinski-Harabasz index (CHI) to evaluate the clustering significance using the R package “fpc.” CHI is the ratio of the sum of between-clusters dispersion and inter-cluster dispersion for all clusters, the higher the score, the better the performances of clustering. To validate the stability of the clustering result, we utilized the bootstrap resampling method in the R package “boot” to randomly resample from the original dataset to generate resampling datasets with the same sample size 1,000 times and calculated the CHIs, respectively, in the same way. Then, a *t*-test was performed to identify if the difference between the CHIs from the resampling datasets and the CHI from the original dataset is statistically significant. The Kaplan-Meier (KM) curves and log-rank tests were used to identify survival differences between the subgroups. Finally, the optimal cluster number was decided by combining the clustering results and the clinical features.

### Immune Landscape of the Subgroups

To estimate the immune infiltration levels of the 28 immune cell types for each BC sample, we performed single-sample gene set enrichment analysis (ssGSEA) to derive an enrichment score for each immune cell population using the R package “GSVA” (18), and the R code and the immune cell maker list were, respectively, shown in Supplementary Tables 1, 7. The enrichment scores were normalized using the Min-Max Normalization method, which turned the scores into values ranging from 0 to 1. The marker gene set for the 28 immune cell types was obtained from a previous study (19), which included data from innate and adaptive immune cells. We used the ESTIMATE algorithm to assess the tumor microenvironment of each BC sample by calculating immune scores, stromal scores, and tumor purity based on the specific gene expression signatures of immune cells and stromal cells.

### Calculation of Tumor Mutation Burden

Tumor mutation burden (TMB) is defined as the total number of non-synonymous mutations per million bases. Missense, non-sense, splice-site, and frameshift mutations were considered non-synonymous for the purposes of this study. We calculated the TMB score using the formula: TMB score = the number of non-synonymous mutations/length of exons (38 million), for each sample (20). Wilcoxon rank-sum tests were used for comparisons between two groups, and Kruskal-Wallis tests were used for three or more groups.

### Differential Analysis and Functional Annotation of the Subgroups

To clarify the functional differences between the subgroups, we first performed differential analyses to identify differentially expressed genes (DEGs) between the subgroups, using the R package “limma” and the gene expression profiles of BC (21). The criteria for identification of DEGs was an adjusted *p* < 0.05 and |Log2 (Fold Change)| > 1. These DEGs were subjected to functional annotation *via* gene ontology (GO) enrichment analysis and the Kyoto Encyclopedia of Genes and Genomes (KEGG) pathway enrichment analysis, using the R package, “clusterProfiler” (22).

### Construction and Validation of the Prognostic Model of IC-SHMS

Multiple Cox proportional hazard models were constructed with different prognostic features using the R package “survival” (23, 24) and the algorithm equation used in the package was displayed in Supplementary Table 2. IC-SHMSs showing independent prognostic values were used to construct the IC-SHMS model. To investigate the efficacy and stability of the IC-SHMS model, we randomly divided BC patients into a training set and a validation set, at a ratio of 7:3. The training set was used to construct the model, while the validation set was used to test the model. Subsequently, the R package “rms” was used to determine the optimal model, in which the algorithm was used to select the optimal combination of IC-SHMSs *via* Akaike's Information Criterion (AIC) in a stepwise approach. The IC-SHMSs included in the final model were used to establish the risk signature, and the risk score for each patient was calculated using the function “predict” supplied in the R package “stats.” Patients were divided into high and low-risk groups based on the median of risk scores. Kaplan-Meier (KM) curves and log-rank tests were used to identify survival differences between the two groups. The area under the curve (AUC) of time-dependent receiver operating characteristic (ROC) curve was calculated to evaluate the prediction accuracy for 3- and 5-year overall survival (OS) using the R package “survivalROC” (25), and the equation used in the package was also shown in Supplementary Table 2. Finally, the IC-SHMS model developed on the training set was applied to the validation set in order to quantify its efficacy and stability.

### Comparison of the Prognostic Value of the IC-SHMSs and Clinical Features

To compare the prognostic value of the IC-SHMSs and clinical features, we used all BC patients' survival information and different prognostic features to, respectively, construct an IC-SHMS model and a clinical model. The IC-SHMS model was constructed using the IC-SHMSs with independent prognostic values as described before, and the clinical model was constructed using the clinical features of patient age and TNM stage. Time-dependent ROC analyses were then performed to estimate the survival prediction accuracy of these two models separately, using 1,000 × bootstrap resampling. Finally, to identify whether the prognoses predicted by the IC-SHMSs could significantly improve the prediction accuracy of the clinical model, we integrated the IC-SHMSs with the clinical features to construct a combined model, and calculated the integrated discrimination improvement (IDI) compared with the clinical model, using the R package “survIDINRI” (26). In constructing each optimal prognostic model, a stepwise algorithm incorporating AIC was used to select the features.

### Clinical Application of the Combined Model

The features in the combined model were used to establish a risk signature, and the risk scores of BC patients were calculated for risk stratification purposes. Kaplan-Meier (KM) curves and log-rank tests were used to identify differences in OS between high- and low-risk groups, defined based on the median of risk scores. A nomogram with weighted scores calculated using the features included in the combined model was used to predict the 3- and 5-year OS of BC patients. Calibration curves were created to test the accuracy of the survival prediction of the combined model compared with that of an ideal model.

### Statistical Analyses

All statistical analyses were conducted using R software, version 3.63 (https://www.r-project.org/). Appropriate R packages and statistical methods were selected for different analyses. The R package “survival” was used for survival analysis, and only patients whose follow-up time was >30 d were included. Wilcoxon rank-sum tests were used for mean comparisons between two groups, and Kruskal-Wallis tests were used for three or more groups. Statistical significance was set at *p* < 0.05.

## Results

### Data Preparation

**Figure 1:**
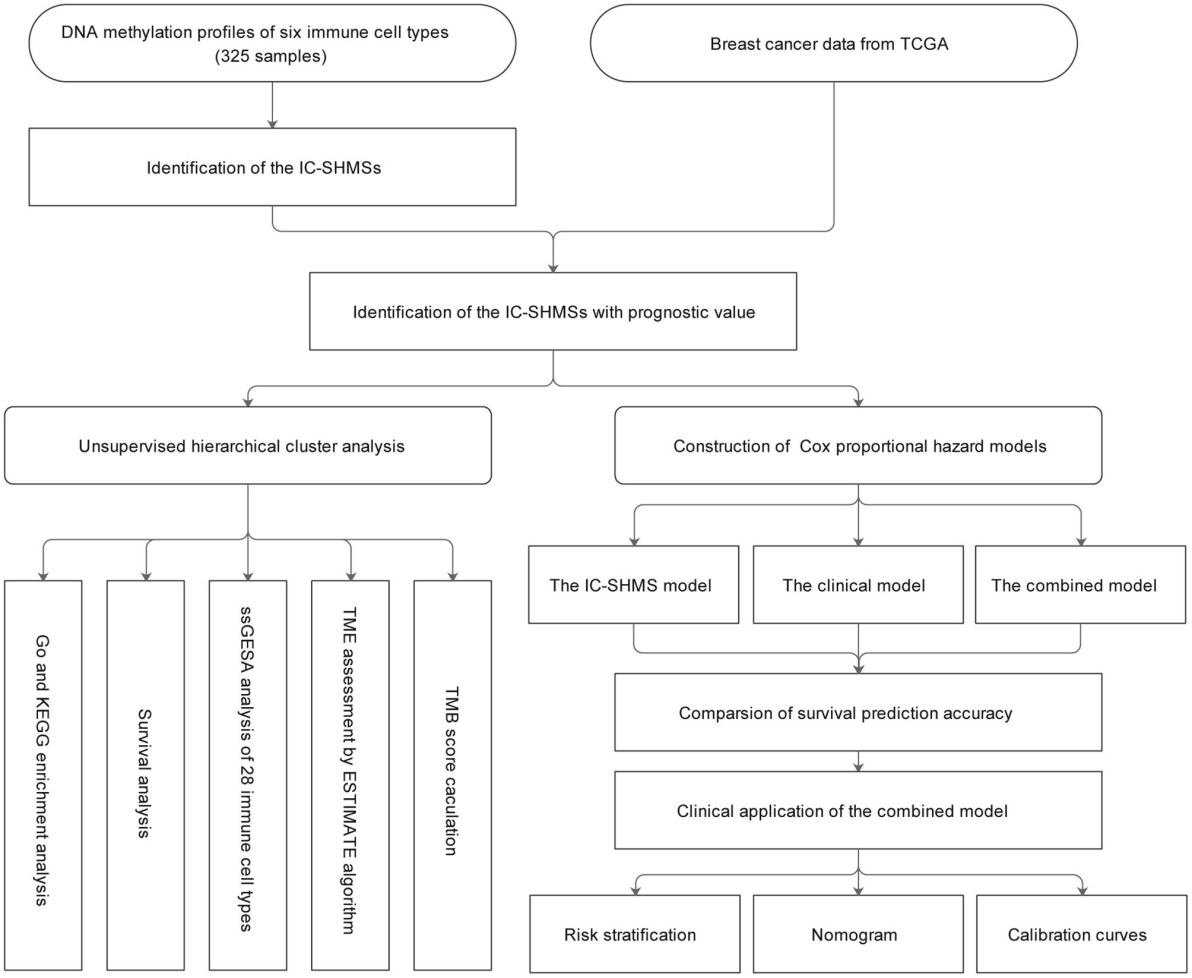
Workflow chart. IC-SHMS, immune cell-specific hypermethylation signatures; Go, gene ontology; KEGG, Kyoto encyclopedia of genes and genomes; ssGSEA, single-sample gene set enrichment analysis; TME, tumor microenvironment; TMB, tumor mutation burden.

A total of 325 samples obtained from six immune cell types (CD14^+^ monocyte: 45; CD19^+^ B cell: 117; CD4^+^ T cell: 94; CD8^+^ T cell: 41; CD56^+^ NK cell: 14; neutrophil: 14) found in eight DNA methylation datasets were used to identify the IC-SHMSs. A methylation beta value matrix containing 774 BC samples was used to detect the prognostic value of the IC-SHMSs and to perform cluster analysis. The gene expression matrix of 1,077 BC samples was used to assess the immune landscape. Somatic mutation data containing 985 BC samples were used to calculate the tumor mutation burden (TMB) score. The entire workflow is shown in Figure 1.

### Identification of the IC-SHMSs

**Figure 2:**
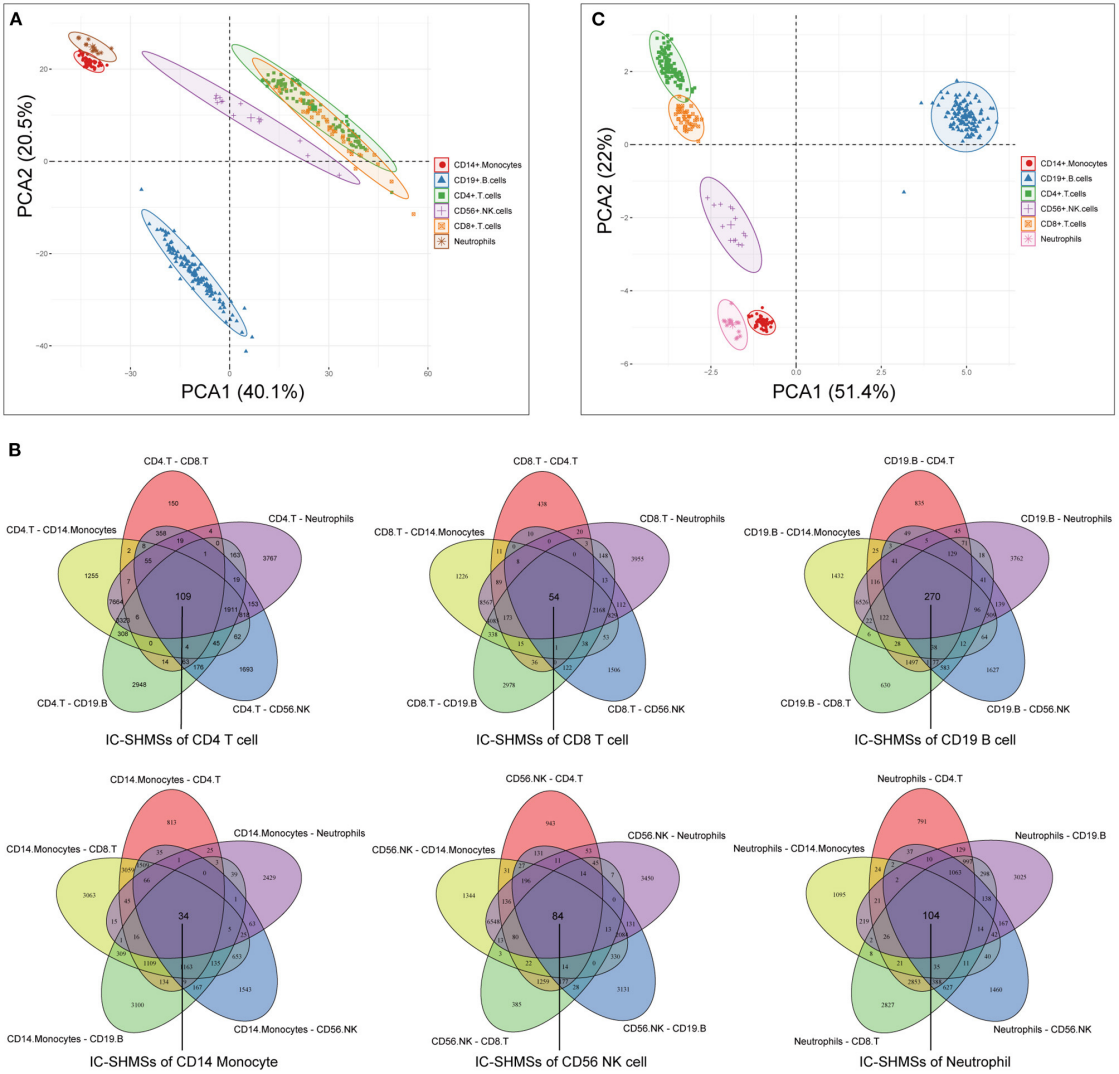
Identification of the IC-SHMSs of six immune cell types. **(A)** PCA plot of six immune cell types based on the whole methylation profiles. **(B)** Venn diagrams showing the IC-SHMS numbers of each immune cell; a total of 655 IC-SHMSs was identified. **(C)** PCA plot of six immune cell types based on the methylation profiles of the 655 IC-SHMSs.

**Figure 3:**
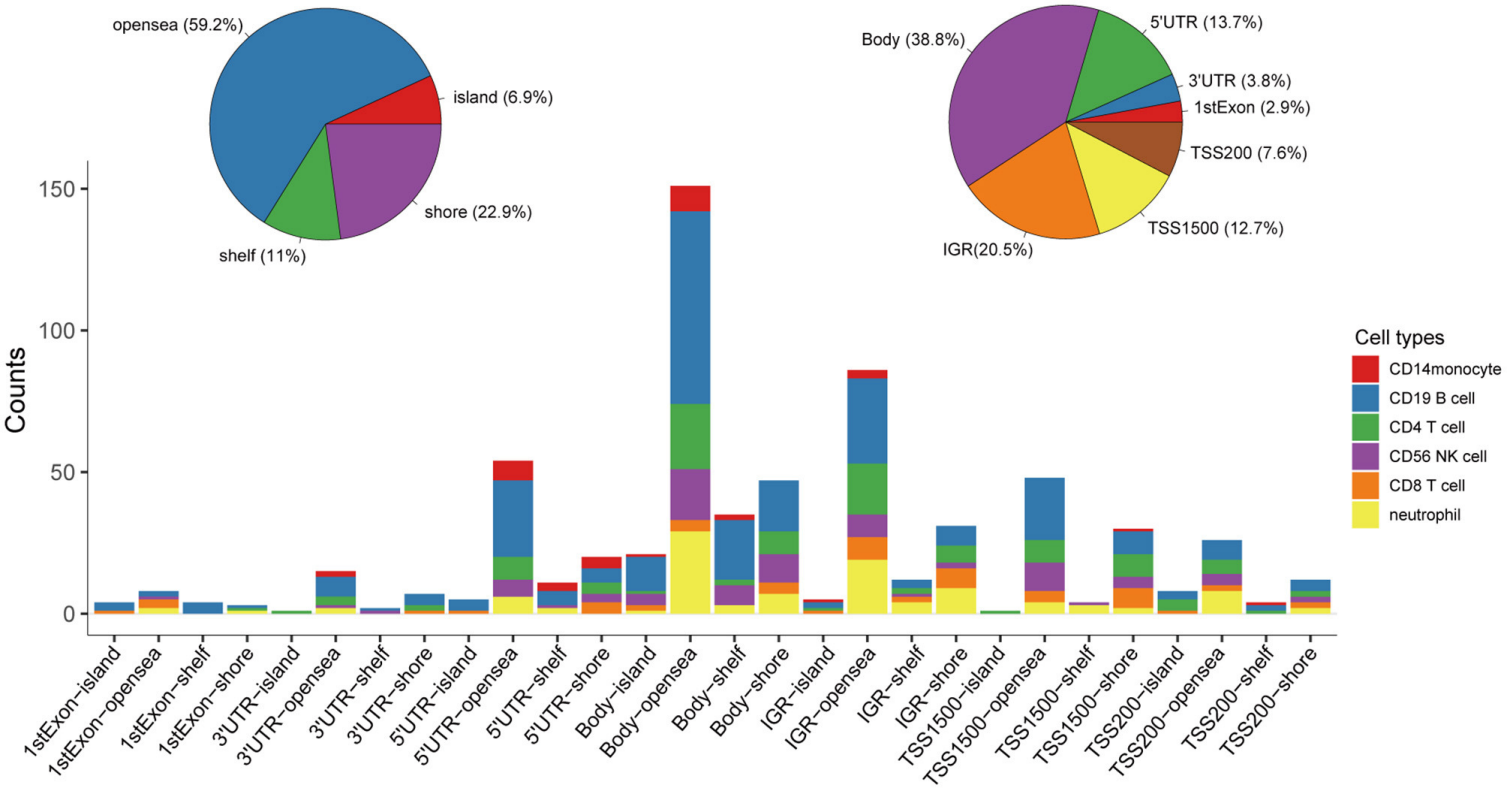
Distributions corresponding to the gene region and CpG island of the IC-SHMSs. Most of these IC-SHMSs were located in the region of gene body and CpG island opensea. IGR, Intergenic region; TSS, transcription start site; UTR, untranslated region.

Following quality control and correction for batch effects in the methylation profiles, the combined methylation beta value matrix contained a total of 325 immune cell samples and 361,262 methylation probes. The PCA plot clearly discriminated between these six types of immune cells (Figure 2A). A total of 655 IC-SHMSs was identified from the six immune cell types as follows: CD14^+^ monocytes, 34; CD19^+^ B cells, 270; CD4^+^ T cells, 109; CD8^+^ T cells, 54; CD56^+^ NK cells, 84; and neutrophils, 104 (Figure 2B; Supplementary Table 3). PCA analysis of immune cells was performed based on the methylation profiles of these 655 IC-SHMSs. The discrimination between immune cells was more obvious than that based on the whole methylation profiles, indicating that IC-SHMSs may accurately represent these immune cells (Figure 2C). The distributions corresponding to the gene region and CpG island of these IC-SHMSs are shown in Figure 3, which shows that IC-SHMSs were most frequently located in the region of gene body and CpG island opensea. In addition, univariate Cox regression analyses suggested that 30/655 IC-SHMSs (CD14^+^ monocyte: 4; CD19^+^ B cell: 12; CD4^+^ T cell: 5; CD8^+^ T cell: 1; CD56^+^ NK cell: 5; and neutrophil: 3), age, and TNM stage may have prognostic values. Those features were used in multivariate Cox regression analyses, and the results showed that 10/30 IC-SHMSs, age, and TNM stage may have independent prognostic values (Table 1). This observation indicated that the IC-SHMSs which showed prognostic value may affect the prognoses of BC patients by regulating immune cell function.

**Table 1:** Statistical summary of the IC-SHMSs with prognostic value.

		**Univariate analysis**		**Multivariate analysis**	
**Item**	**Cell.type**	**HR (95% CI)**	***p*-value**	**HR (95% CI)**	***p*-value**
age	–	1.04 (1.02–1.05)	<0.001	1 (1–1.1)	<0.001
TNM stage II	–	1.98 (0.93–4.21)	0.077	1.6 (0.71–3.6)	0.262
TNM stage III	–	3.35 (1.54–7.27)	0.002	3.8 (1.7–8.7)	0.001
TNM stage IV	–	29.0 (10.68–78.9)	<0.001	46 (15–140)	<0.001
cg08708961	Neutrophil	2.2 (1.1–4.1)	0.019	5.5 (2–15)	<0.001
cg19473529	CD4.T.cell	0.28 (0.08–0.94)	0.039	0.05 (6.4 e-03–0.31)	0.002
cg24536818	CD19.B.cell	0.28 (0.1–0.78)	0.015	0.1 (0.02–0.43)	0.002
cg24088496	CD56.NK	3.6 (1.5–8.9)	0.005	6.3 (1.8–22)	0.004
cg17124583	CD19.B.cell	6.5 (1–41)	0.045	20 (2–210)	0.011
cg17988310	CD56.NK	0.35 (0.16–0.78)	0.01	0.22 (0.07–0.74)	0.014
cg14084689	CD56.NK	10 (1.6–67)	0.014	12 (1.6–88)	0.015
cg10639435	CD14.monocyte	6.1 (1.4–27)	0.017	12 (1.4–110)	0.023
cg11930955	CD56.NK	8.4e-05 (1.1e-07–0.06)	0.005	2.5e-06 (7.6e-12–0.82)	0.0465
cg07141504	CD4.T.cell	0.02 (2.5e-04–0.18)	<0.001	0.004 (1.9e-05–0.99)	0.0498
cg21278103	CD19.B.cell	0.29 (0.12–0.7)	0.005		
cg16704703	CD19.B.cell	0.13 (0.02–0.72)	0.02		
cg25869889	CD19.B.cell	0.22 (0.07–0.67)	0.008		
cg06470558	Neutrophil	0.06 (3.6e-03–0.87)	0.04		
cg01151584	CD14.monocyte	9.5 (1.3–71)	0.029		
cg01946401	CD4.T.cell	0.2 (0.06–0.69)	0.011		
cg18445438	CD19.B.cell	0.08 (0.01–0.45)	0.005		
cg07240557	CD19.B.cell	0.38 (0.15–0.98)	0.046		
cg22843803	CD14.monocyte	0.08 (8.5e-03–0.67)	0.021		
cg26687579	CD56.NK	0.06 (7.3e-03–0.51)	0.01		
cg11658419	CD8.T.cell	0.19 (0.04–0.87)	0.032		
cg04136456	CD4.T.cell	0.16 (0.04–0.68)	0.013		
cg02493211	CD19.B.cell	0.12 (0.02–0.83)	0.032		
cg11906021	Neutrophil	0.08 (0.01–0.63)	0.017		
cg14655843	CD14.monocyte	7.9 (1.5–42)	0.015		
cg13437525	CD4.T.cell	0.004 (3.5e-05–0.48)	0.024		
cg06881965	CD19.B.cell	0.26 (0.09–0.77)	0.014		
cg24171555	CD19.B.cell	0.12 (0.02–0.77)	0.026		
cg23925650	CD19.B.cell	0.3 (0.11–0.82)	0.018		
cg20541456	CD19.B.cell	0.27 (0.09–0.79)	0.017		

*Only the items with p < 0.05 were displayed. TNM stage I was defined as the reference of stage II, stage III, and stage IV. IC-SHMS, immune cell-specific hypermethylation signature; HR, hazard ratio; CI, confidence interval*.

### Identification of the Subgroups of BC Patients

**Figure 4:**
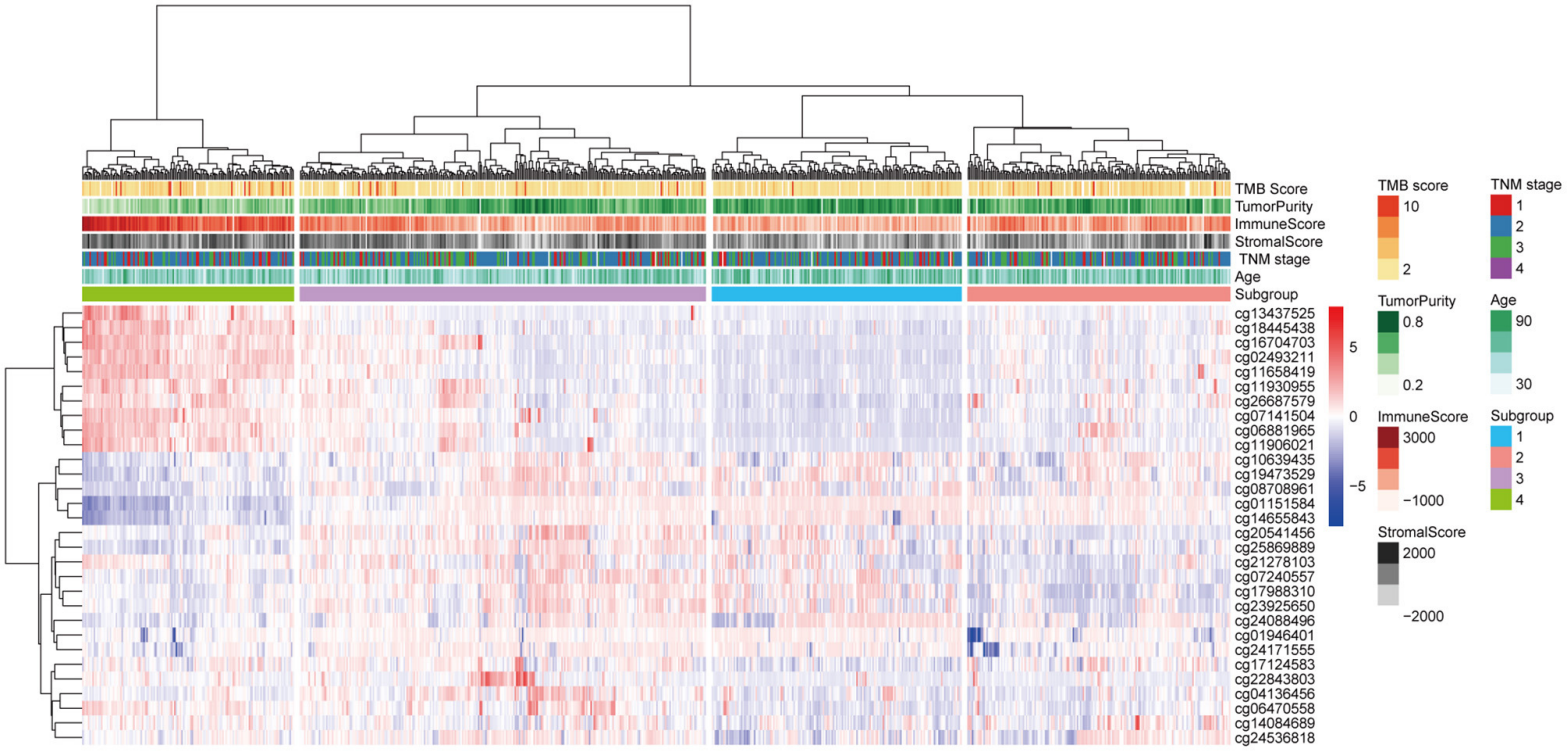
Heatmap showing the distribution of the beta values of the 30 IC-SHMSs with prognostic value between the four subgroups.

**Figure 5:**
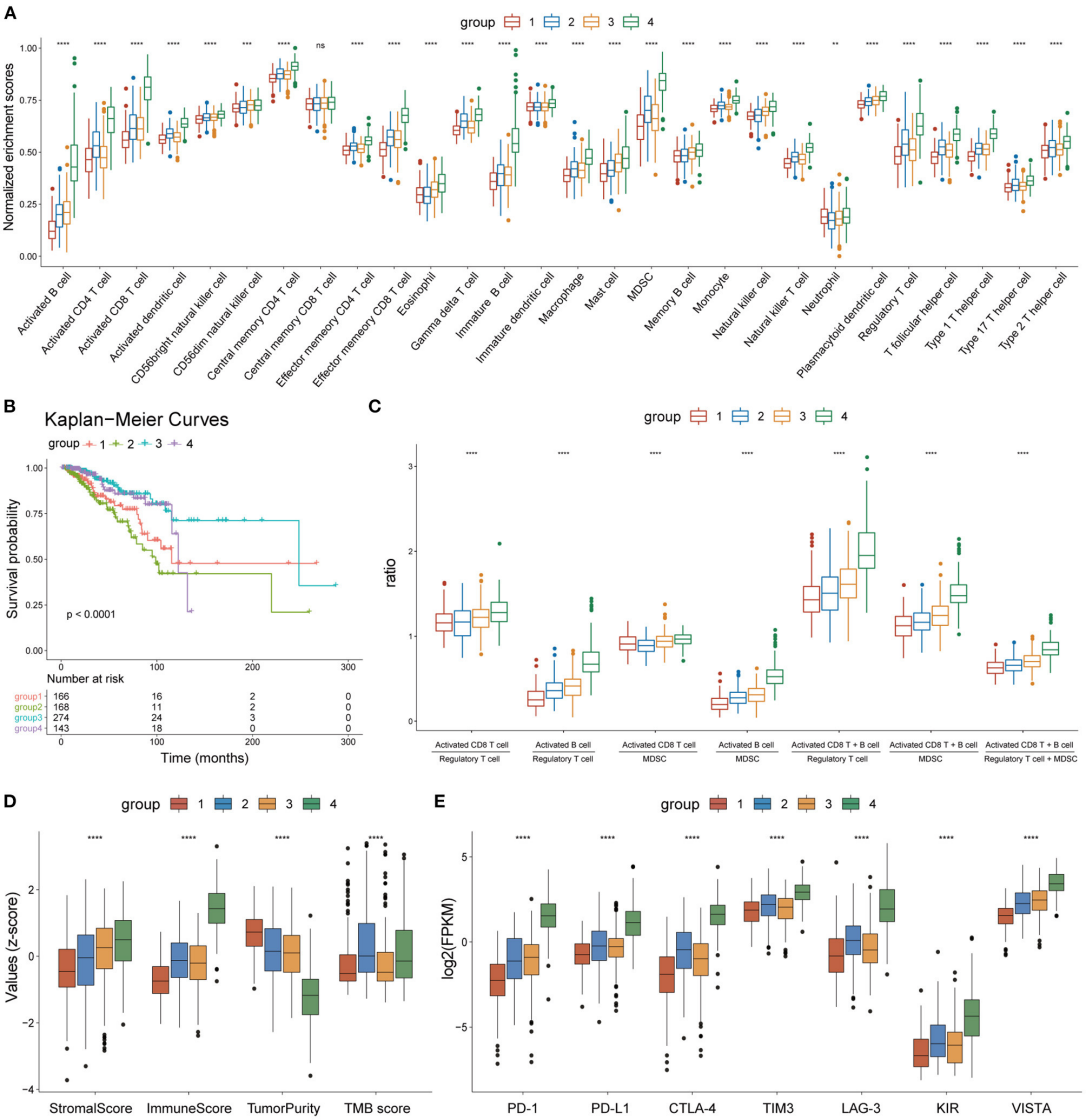
Heterogeneity of the four subgroups. **(A)** Box plot showing enrichment scores of 28 immune cell types between the four subgroups. **(B)** Overall survival curves of the four subgroups. **(C)** Box plot showing the ratio of immune activation to immune suppression in the four subgroups. **(D)** Box plot showing the distribution of immune scores, stromal scores, tumor purity, and TMB scores in the four subgroups, The scores of each character were normalized by the *z*-score method. **(E)** Box plot showing the expression levels of seven immune checkpoint molecules. ^*^p < 0.05, ^**^p < 0.01, ^***^p < 0.001, ^****^p < 0.0001.

The 30 IC-SHMSs showing prognostic value were used as variables to perform clustering analysis and four subgroups of BC patients were identified. Besides, the results of the CHI analyses showed the clustering has a good significance when k = 4 (CHI = 98.23; Supplementary Table 4), and the *t*-test result indicated the difference between the CHI distribution from the resampling datasets and the CHI value from the original dataset is not statistically significant (*p* = 0.1662; Supplementary Figure 1). A heatmap of the beta values of the 30 IC-SHMSs is displayed in Figure 4. It indicates significant differences in the methylation levels of IC-SHMSs between different groups. In addition, survival analysis showed that patients in group 3 and group 4 had a significantly better OS than patients in group 1 and group 2 (Figure 5B). Therefore, our findings indicate that IC-SHMSs can be used to effectively group BC patients.

### Immune Landscape and TMB Score Distribution of the Four Subgroups

The enrichment scores of the 28 immune cell types were calculated for each BC sample using the ssGSEA method and were normalized using the Min-Max Normalization method. The distribution of enrichment scores of the 28 immune cell types between the four subgroups is shown in Figure 5A. Group 1 had the lowest immune response; group 2 showed moderate immune activation and relatively high immune suppression; group 3 displayed medium immune activation and relatively low immune suppression; group 4 showed the strongest immune response. Combined with the results of survival analysis, these results indicate that the ratio of immune activating cells (activated B cells and activated CD8 T cells) to immune suppressing cells [myeloid-derived suppressor cells (MDSCs) and regulatory T cells] may play an important role in tumor progression and patient survival. A box plot depicting the ratio of immune-activating cells to immune-suppressing cells of the four subgroups strongly supported this hypothesis (Figure 5C).

On the other hand, the tumor microenvironment of each BC sample was assessed using immune scores, stromal scores, and tumor purity, using the ESTIMATE algorithm. The distribution of these features between the four subgroups showed significant differences (Figure 5D). TMB scores were calculated as previously described, and outliers detected by the quartile method were deleted. The box plot showed that the TMB scores of groups 2 and 4 were significantly higher than those of groups 1 and 3 (Figure 5D). A box plot of the expression levels of seven immune checkpoint molecules (PD-1, PD-L1, CTLA-4, TIM3, LAG3, KIR, and VISTA) also showed significant differences between the four subgroups (Figure 5E). Thus, our findings indicate that the four subgroups displayed heterogeneity in immune landscapes and somatic mutations.

### Differential Analysis and Functional Annotation of the Subgroups

**Figure 6:**
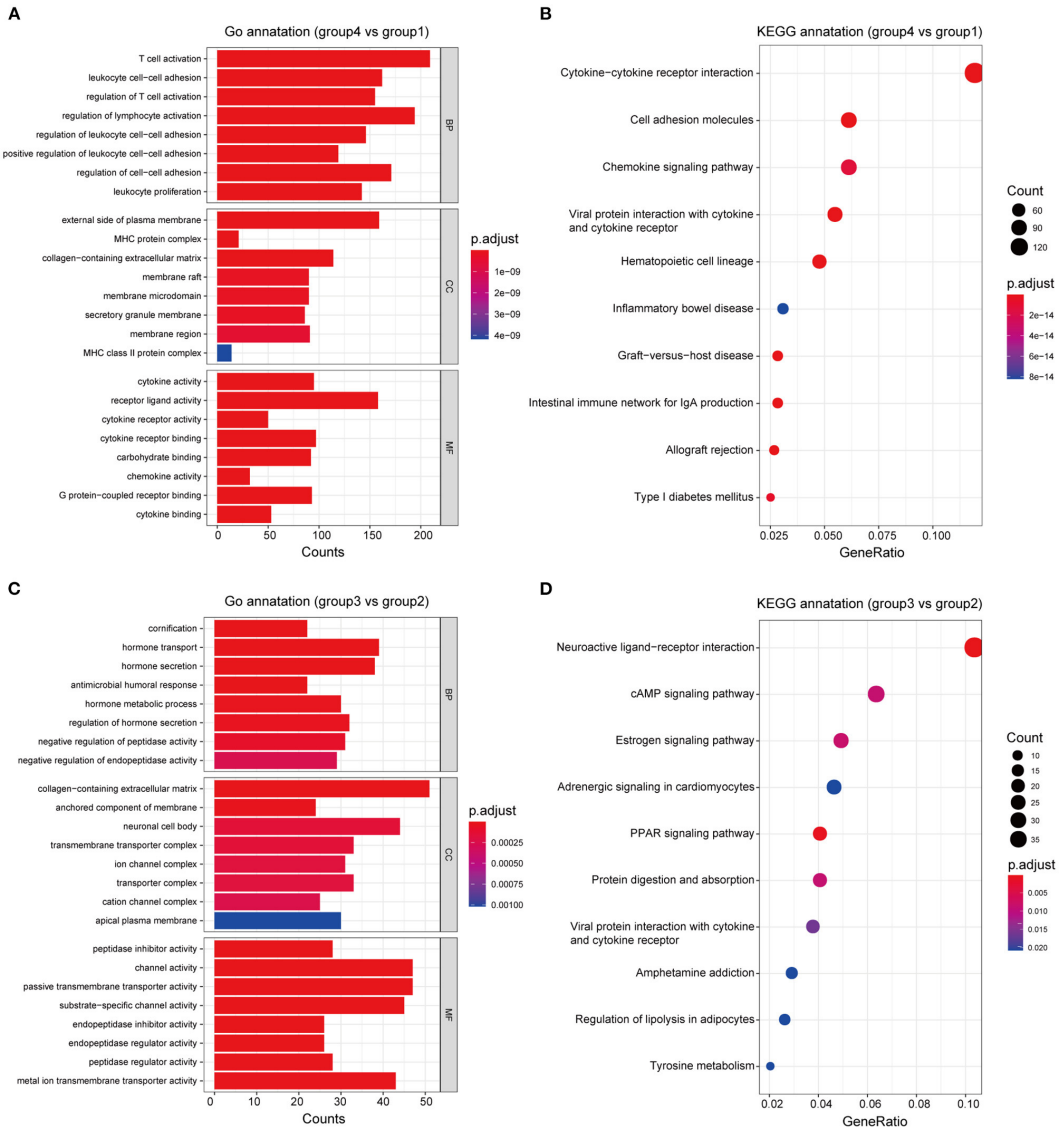
Functional annotation of the subgroups. GO and KEGG enrichment analyses, respectively, revealed the significantly associated processes and signaling pathways with differences in immune response between groups 4 and 1 **(A,B)**, and between groups 3 and 2 **(C,D)**.

Based on differences in the immune landscape between the subgroups, we performed two differential analyses: group 4 vs. group 1; and group 3 vs. group 2, resulting in 2,849 and 865 significant DEGs, respectively, being identified (Supplementary Figure 2). GO and KEGG enrichment analyses indicated that the difference in immune response between group 4 and group 1 may be closely associated with the biological processes of lymphocyte activation and leukocyte cell-cell adhesion, and that the signaling pathways of cytokine-cytokine receptor interaction, viral protein interaction with cytokines and cytokine receptors and cell adhesion molecules may play an important role in this process (Figures 6A,B; Supplementary Table 5). On the other hand, the difference in immune response between group 3 and group 2 may be closely related to the biological processes of cornification and regulation of hormone secretion, the neuroactive ligand-receptor interaction signaling pathway, the PPAR signaling pathway and the estrogen signaling pathway, all of which may play an important role in this process (Figures 6C,D; Supplementary Table 6). Therefore, these results may clarify the differences in immune responses that were observed between groups, as well as provide clues that are useful for personalized treatment.

### Construction and Validation of the Prognostic Model of IC-SHMS

**Figure 7:**
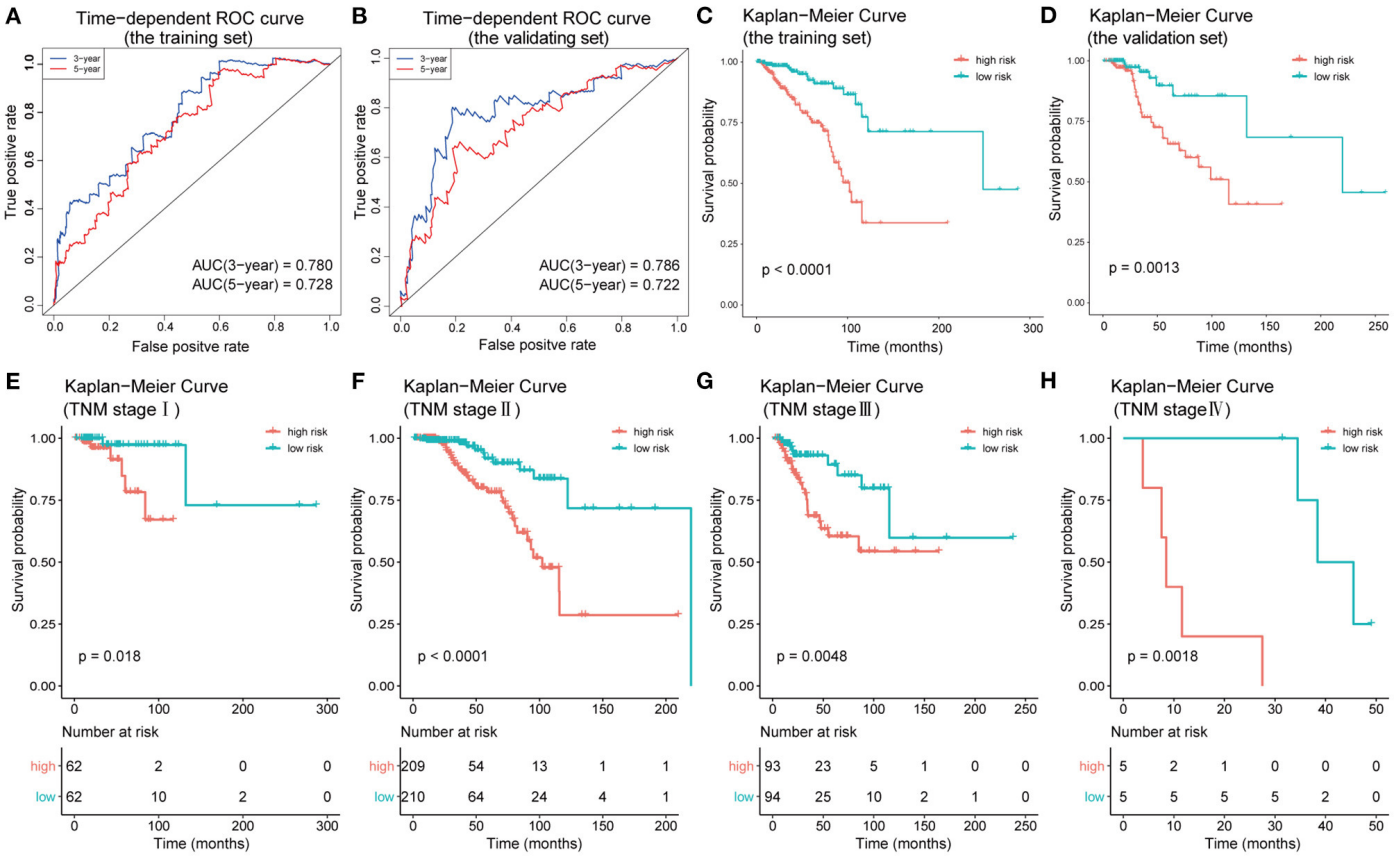
Construction and validation of the prognostic model constructed using IC-SHMSs. Time-dependent ROC analyses for 3- and 5-year OS, respectively, were performed to estimate the prediction accuracy of the prognostic model, using a training set **(A)** and a validation set **(B)**. Overall survival curves of the low- and high-risk groups in the training set **(C)** and the validation set **(D)**. Overall survival curves of the low- and high-risk groups based on the BC patients in TNM stage I, II, III, and IV **(E–H)**.

To test the efficacy of the prognostic model of IC-SHMS, 751 BC patients with follow-up times >30 d were divided into two groups, at a ratio of 7:3, as follows: a training set (*n* = 523); and a validation set (*n* = 228). The training set was used to construct an IC-SHMS model, while the validation set was used to verify the IC-SHMS model. Ten IC-SHMSs showing independent prognostic values were used to construct the Cox proportional hazard model using the training set, and 9/10 IC-SHMSs (cg08708961, cg19473529, cg24088496, cg24536818, cg17124583, cg17988310, cg10639435, cg14084689, and cg07141504) were retained in the optimal model selected by the stepwise algorithm (Supplementary Figure 3). Based on these nine risk signatures in this model, risk scores were calculated for each BC patient in the training set and validation set separately. Time-dependent ROC analyses indicated that both the training set (3-year AUC: 0.780; 5-year AUC: 0.728) and the validation set (3-year AUC: 0.786; 5-year AUC: 0.722) of this model showed similarly good performances in terms of survival prediction accuracy (Figures 7A,B). Patients with high-risk scores had poorer prognoses than those with low-risk scores in the training set, a result which was verified using the validation set (Figures 7C,D). Based on the median risk scores, patients included in the four groups (TNM stages I, II, III, and IV) could be, respectively, stratified into two groups with significantly different prognoses, indicating that higher risk scores were associated with poorer survival (Figures 7E–H). Therefore, our findings demonstrated that the prognostic model based on IC-SHMSs showed good stability and accuracy of survival prediction in BC.

### Comparison of Prediction Accuracy of the IC-SHMS Model, the Clinical Model, and the Combined Model

**Figure 8:**
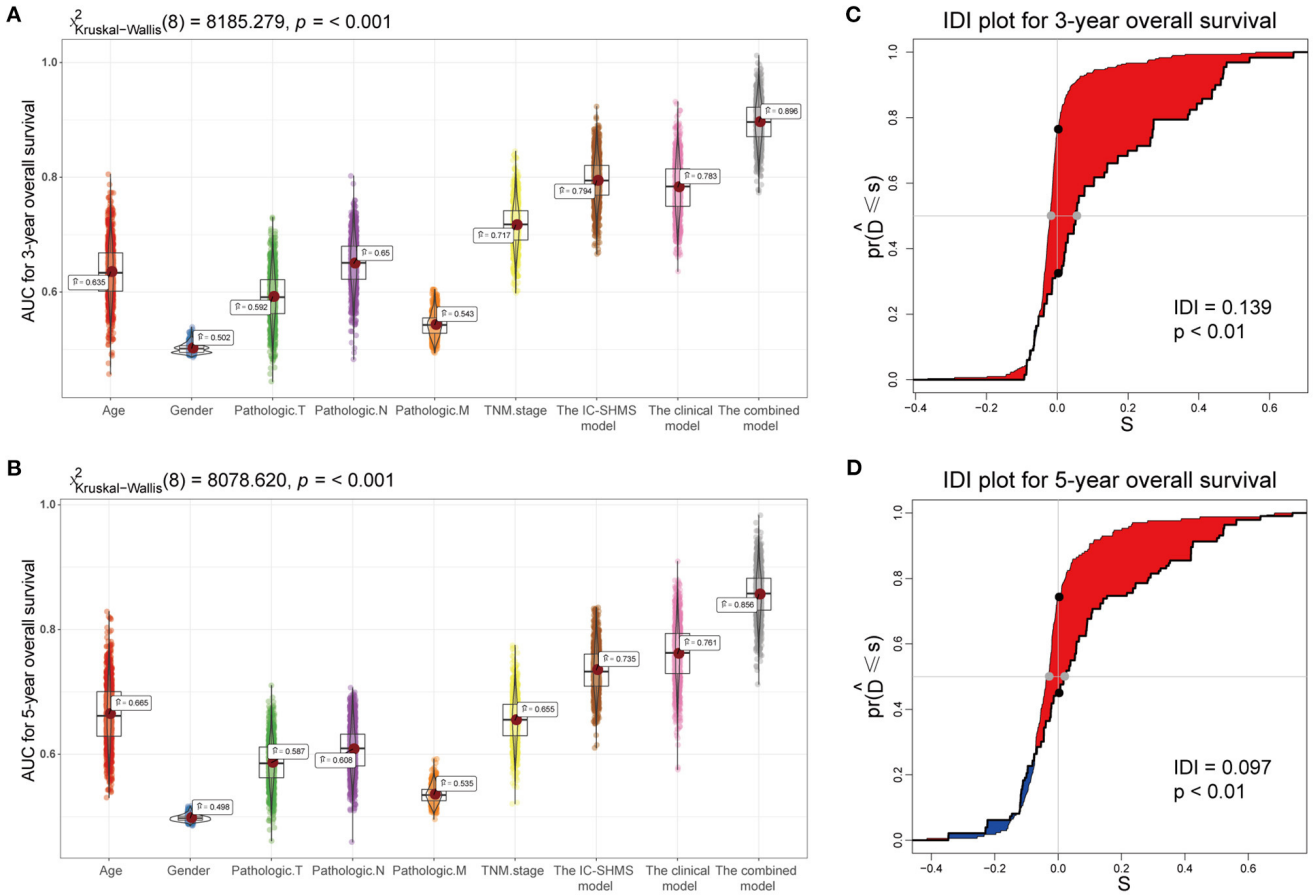
Comparison of the predictive accuracy of the IC-SHMS, clinical, and combined models. **(A,B)** Box plot showing the prediction accuracy for 3- and 5-year OS, based on the AUC with 1,000 × bootstrap resampling. **(C,D)** IDI charts showing that, compared with the clinical model, the combined model with nine additional IC-SHMSs improved the prediction accuracy of 3- and 5-year OS in a statistically significant manner (*p* < 0.05).

**Figure 9:**
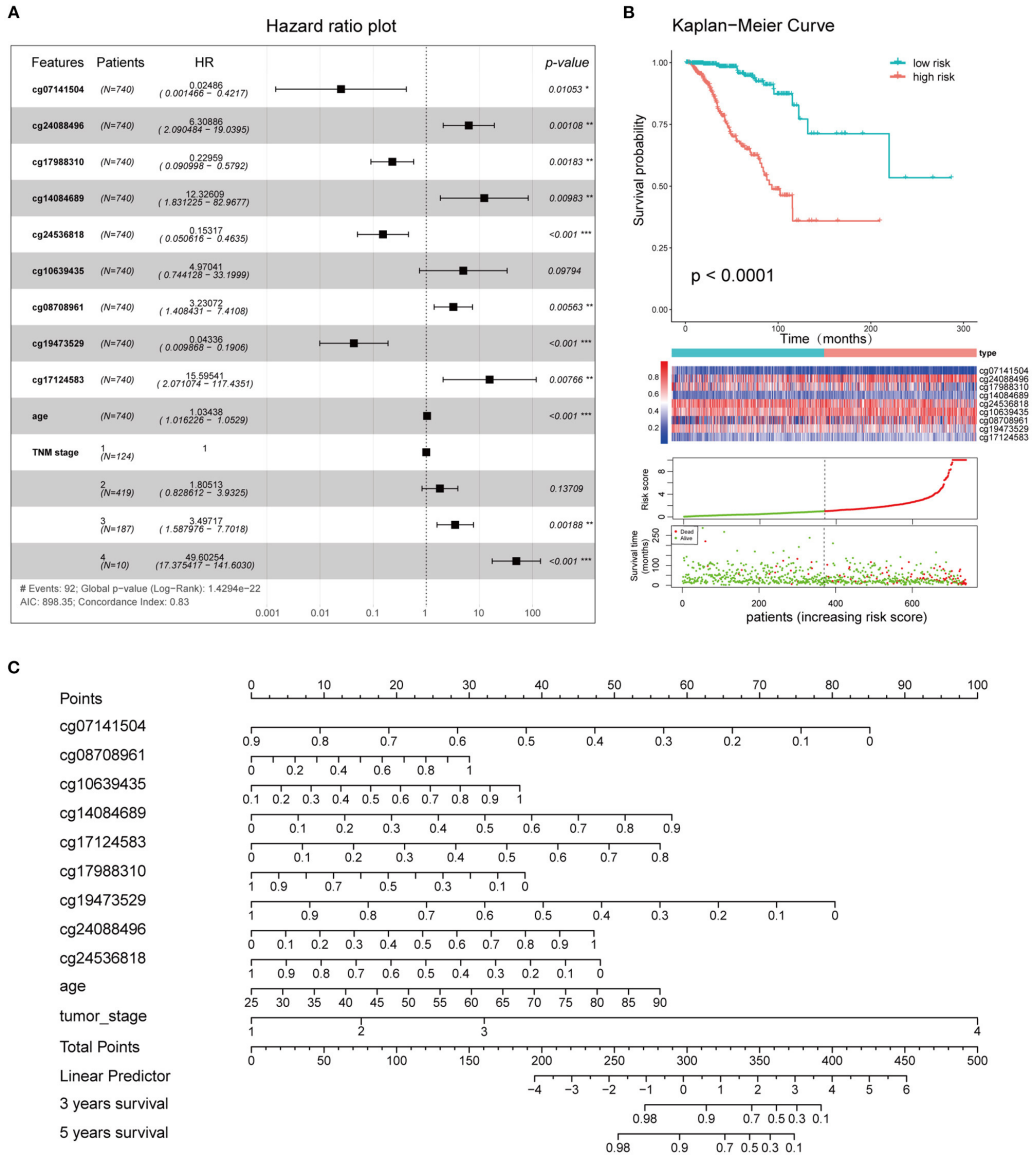
Clinical application of the combined model. **(A)** Survival curves of high- and low-risk groups. **(B)** Forest plot showing the hazard ratios of the risk features included in the combined model. **(C)** Nomogram created using the 11 risk features incorporated into the combined model, to predict the 3- and 5-year OS of BC patients.

Using the survival information and different features of 751 BC patients, three Cox proportional hazard models were constructed as follows: the 10 IC-SHMSs with independent prognostic value were used to construct an IC-SHMS model, and 9/10 IC-SHMSs were selected for the optimal model using AIC (Supplementary Figure 4A); the clinical model was constructed using the clinical features of patient age and TNM stage, both of which features were retained in the optimal model (Supplementary Figure 4B); the combined model was constructed using the 10 IC-SHMSs, age, and TNM stage. 9/10 IC-SHMSs, age, and TNM stage were retained in the optimal model selected using AIC (Figure 9A). Time-dependent ROC analyses showed that the prediction accuracy for 3- and 5-year OS obtained *via* the IC-SHMS model (3-year AUC: 0.793; 5-year AUC: 0.735) was similar to that of the clinical model (3-year AUC: 0.785; 5-year AUC: 0.761), and considerably better than the prediction accuracy obtained using age alone or TNM stage alone. The combined model, with added IC-SHMSs, significantly improved upon the prediction accuracy of the clinical model (3-year AUC: 0.897; 5-year AUC: 0.856); (Figures 8A,B). The IDI results indicated that the improvement that was gained in the accuracy of predicting both 3- and 5-year OS using the combined model was statistically significant when compared with the clinical model (Figures 8C,D). Therefore, our results indicated that IC-SHMSs exhibit good prognostic value and show potential for use as a supplement in the current risk stratification system.

### Clinical Application of the Combined Model

The 11 features (cg08708961, cg19473529, cg24088496, cg24536818, cg17124583, cg17988310, cg10639435, cg14084689, cg07141504, age, and TNM stage) that were included in the combined model were used to establish the risk signatures, following which the risk score of each BC patient was calculated for risk stratification purposes. The hazard ratios associated with these features are shown in Figure 9A. Kaplan-Meier curves and log-rank tests showed a significant difference in OS between the high and low-risk groups as defined based on the median of risk scores (Figure 9B). A nomogram plot was used to predict the 3- and 5-year OS of BC patients, based on the weighted scores of the 11 features incorporated into the combined model (Figure 9C). The calibration curves showed that the combined model had good performance in predicting the 3- and 5-year OS, compared with the ideal model (Supplementary Figure 5).

## Discussion

DNA methylation plays a central role in immune cell differentiation and function, by stabilizing and driving gene activity (27). Both innate and adaptive immune cells in blood are able to infiltrate tumor tissues to form the tumor immune microenvironment (TIME). Previous studies have shown that the TIME, which is heterogeneous and consists of a variety of different immune cells, plays an important role in tumor progression and patient survival-outcomes which are closely associated with immune evasion (28). Tools such as ESTIMATE, TIMER, and CIBERSORTx, which are based on the transcriptomic signatures of immune cells, have been created to assess the status of the TIME. The indicators generated by them have been used for classification and risk stratification in various cancers (29–31). However, few studies have either investigated the methylation signatures of immune cells or linked these signatures with classification and risk stratification in cancer patients. The current study successfully identified the immune cell-specific hypermethylation sites (IC-SHMSs) of six immune cell types separately by analyzing methylome data, and subsequently, applied these IC-SHMSs to classification and risk stratification in BC. Our findings indicated that BC patients could be classified into four heterogeneous clusters based on the 30 IC-SHMSs with prognostic value. Our results indicated that incorporating IC-SHMSs with independent prognostic value into a prognostic model with age and TNM stage may significantly improve the accuracy of prediction of prognosis in BC patients.

BC is a heterogeneous disease, and has significant variability in clinical presentation, response to treatment, and survival prognosis. At an individual level, accurate classification may provide valuable predictive information and guide the selection of patients for adjuvant hormonal therapy, chemotherapy, and radiotherapy (32). The classification of BC according to the expression of estrogen receptors (ER), progesterone receptors (PR), and HER2 is currently standard practice for histopathological examination of BC patients (33). However, this method of classification provides limited information for decision-making related to immunotherapy. Therefore, the development of better predictive biomarkers may facilitate the identification of particular subsets of patients that are most likely to benefit from immunotherapy, either alone or in combination with chemotherapy or other therapies. In this study, four heterogeneous clusters based on IC-SHMSs were identified in BC. These subgroups displayed obvious differences in immune landscapes and survival prognoses. Our findings suggested that the balance between immune activation (activated B cells and activated CD8 T cells) and immune suppression (MDSC and regulatory T cells) was closely related to survival prognosis. This conclusion was consistent with those of previous studies (34, 35), which showed that the ratio of CD8^+^ T cells to regulatory T cells plays an important role in the survival prognosis and molecular subtypes of BC patients. Furthermore, our findings may provide important indicators for personalized immunotherapy. For instance, patients in group 1, characterized by the lowest immune response, lowest expression levels of all seven immune checkpoint molecules, highest tumor purity and poor prognoses, may benefit from a combination therapy of enhanced immunity and targeted tumor cell, while patients in group 2, characterized by medium immune activation, relatively high immune suppression, relatively high expression levels of immune checkpoint molecules of CTLA-4 and LAG-3 and poor prognoses, may benefit from a combination therapy of enhanced immunity and immune checkpoint inhibitors. Many studies have shown that, compared with monotherapy, a combination of immunotherapy with conventional therapies such as chemotherapy and radiotherapy, may significantly improve cancer therapeutic efficacy, possibly leading to the development of promising methods of treatment (36–38). We propose that combining the traditional classification method of BC with our classification method would lead to BC patients being offered more accurate and personalized treatment plans.

In addition to exploring the differences in immune landscapes that were observed between subgroups, we performed GO and KEGG enrichment analyses to perform functional annotation of these differences with respect to immunity. Our results suggested that the differences in immune response between groups 4 and 1, which were associated with immune activation, centered on the processes of regulating lymphocyte activation and leukocyte cell-cell adhesion, as well as cytokine-cytokine receptor interaction signaling. Our results also indicated that a few hormone-related biological processes and signaling pathways, such as hormone secretion and transport, and the estrogen signaling pathway, may be closely related to the differences in immune suppression between groups 3 and 2. Although estrogen and its metabolites are known to be important to the development of BC, the association and mechanism between estrogen and immunity remains unclear (39, 40). Our findings regarding the regulatory relationship between hormones and immune suppression are consistent with those of several previous studies. For instance, Svoronos et al. (41) showed that estrogens facilitated tumor progression by driving MDSC mobilization and augmenting their immunosuppressive activity. Segovia-Mendoza et al. (42) reported that functions in, and responses of, infiltrating immune cells in BC are regulated by steroid hormones and their receptors. Thus, our study identified several major biological processes, as well as key signaling pathways associated with the variability in immune responses displayed by these subgroups, thereby providing important guidelines for personalized treatment.

As understanding of the association between the TIME and tumor progression improves, immunotherapy has also rapidly advanced. To date, immunotherapies using PD-1/PD-L1 or CTLA-4 antagonistic antibodies have shown promising outcomes in various cancers such as melanoma, liver cancer and non-small cell lung cancer, thereby establishing immunotherapy as one of the most promising new therapeutic approaches (43–45). However, due to the heterogeneity of the TIME, and the effects of various resistance mechanisms, <25% of patients treated with immunotherapy have shown a meaningful response (46). Therefore, efforts to identify other specific, effective biomarkers that may be used for immunotherapy are important. In this study, 30 IC-SHMSs with prognostic value, closely related to the identity and/or function of immune cells and potential targets of immunotherapy, were identified. The dynamic plasticity of the epigenome makes it susceptible to therapeutic operations, and the past few years have witnessed an unprecedented development of targeted epigenetic therapies. The most clinically advanced epigenetic therapies in cancers thus far are DNA hypomethylating agents, such as DNA methyltransferase inhibitors, which restore aberrant hypermethylation patterns to the normal phenotype, and thereby provide significant therapeutic advantages compared to genetic alterations (47). Preclinical studies suggest that DNA methyltransferase inhibitors have the greatest efficacy when combined with other cancer therapies. Although epigenetic therapy is undoubtedly a potential and powerful tool that is especially associated with immunity based cancer therapy, much work is required to produce satisfactory treatments.

Currently, risk stratification of BC patients is predominantly based on clinicopathological characteristics, and the TNM risk stratification system remains the gold standard. However, an increasing number of studies have shown that many other characteristics may be used to supplement the TNM system. For instance, Mavaddat et al. (48) assessed the role of genetic variants in risk stratification of BC patients. Lai et al. (49) incorporated novel miRNAs into a prognostic model for BC patients. CD8^+^ T cell and NK cell infiltration has been shown to serve as an independent prognostic biomarker of BC (34, 50). An increasing number of tumor-related methylation markers are being discovered in BC (51), and Tao et al. (52) has proposed a seven DNA methylation signature-based prognostic model. The current study explored the role of specific methylation signatures of immune cells in the risk stratification of BC patients. Compared with Tao's seven methylation signature prognostic model, the IC-SHMS model with nine methylation signatures of immune cells used in our study yielded a more accurate survival prediction (AUC: 0.793 vs. 0.74). Besides, adding these IC-SHMSs to the clinical model of patient age and TNM stage significantly improved the accuracy of prognosis prediction (AUC: 0.897). Although our results indicated the IC-SHMS model almost has the same predictive efficacy for prognosis as the clinical model, it doesn't exceed. We think possible reasons leading to this result include: firstly, these clinical prognostic markers are selected through long-term clinical practice, so its predictive efficacy for prognosis is very reliable; secondly, the tumor microenvironment is a complex regulatory system composed of a highly heterogeneous population of cancer cells, as well as a large variety of resident and infiltrating host cells, extracellular matrix proteins, and secreted proteins. Although TIME plays an important role in tumor progression, it is just a small part of the whole tumor microenvironment. Therefore, the predictive efficacy for prognosis is limited to a certain extent by only using the IC-SHMSs as prognostic factors and many other molecular characteristics also should be considered. Therefore, our findings suggest that IC-SHMSs may be used not only for classification but also to supplement the TNM risk stratification system.

To our knowledge, ours is the first study to correlate immune cell hypermethylation signatures with classification and risk stratification in BC. Our finding of four subgroups and 30 IC-SHMSs with prognostic value may contribute to personalized treatment and targeted therapy in BC patients, and the new prognostic model constructed in this study is expected to increase the accuracy of risk stratification, thereby contributing to decisions pertaining to clinical treatment that may lead to improved outcomes for BC patients. However, the volume of data used in this study was limited, and these findings may require more clinical data and experiments in order to be fully validated. Our future research entails testing additional clinical data and performing additional mechanistic experiments on the signatures that have been identified.

In conclusion, our study demonstrated that IC-SHMSs are well-suited to serve as signatures of classification and risk stratification in BC. Furthermore, this study provides new insights into the use of epigenetic signatures, which may help improve subtype identification, risk stratification, and the management of treatment.

## Data Availability Statement

The datasets presented in this study can be found in online repositories. The names of the repository/repositories and accession number(s) can be found in the article/Supplementary Materials.

## Author Contributions

This study was performed in collaboration among all authors. YC and XL contributed to the study design. YC, BJ, and WW downloaded the datasets and performed the statistical analyses. YC and FX analyzed the results. YC drafted the manuscript. All authors contributed to revision of the final manuscript. XL supervised the study.

## Conflict of Interest

The authors declare that the research was conducted in the absence of any commercial or financial relationships that could be construed as a potential conflict of interest.

## Publisher's Note

All claims expressed in this article are solely those of the authors and do not necessarily represent those of their affiliated organizations, or those of the publisher, the editors and the reviewers. Any product that may be evaluated in this article, or claim that may be made by its manufacturer, is not guaranteed or endorsed by the publisher.

## References

[1] Cardoso F Harbeck N Barrios CH Bergh J Cortés J El Saghir N. Research needs in breast cancer. Ann Oncol. (2017) 28:208–17. 10.1093/annonc/mdw571 27831505

[2] Garrido-Castro AC Lin NU Polyak K. Insights into molecular classifications of triple-negative breast cancer: improving patient selection for treatment. Cancer Discov. (2019) 9:176–98. 10.1158/2159-8290.CD-18-1177 30679171 PMC6387871

[3] Ernst B Anderson KS. Immunotherapy for the treatment of breast cancer. Current Oncol Rep. (2015) 17:5. 10.1007/s11912-014-0426-9 25677118

[4] Laplagne C Domagala M Le Naour A Quemerais C Hamel D Fournié JJ. Latest advances in targeting the tumor microenvironment for tumor suppression. Int J Mol Sci. (2019) 20:4719. 10.3390/ijms20194719 31547627 PMC6801830

[5] Zhou R Zhang J Zeng D Sun H Rong X Shi M. Immune cell infiltration as a biomarker for the diagnosis and prognosis of stage I–III colon cancer. Cancer Immunol Immunother. (2019) 68:433–42. 10.1007/s00262-018-2289-7 30564892 PMC6426802

[6] Wang H Wu X Chen Y. Stromal-immune score-based gene signature: a prognosis stratification tool in gastric cancer. Front Oncol. (2019) 9:1–14. 10.3389/fonc.2019.01212 31781506 PMC6861210

[7] Bense RD Sotiriou C Piccart-Gebhart MJ Haanen JBAG Van Vugt MATM De Vries EGE. Relevance of tumor-infiltrating immune cell composition and functionality for disease outcome in breast cancer. J Natl Cancer Inst. (2017) 109:djw192. 10.1093/jnci/djw192 27737921 PMC6284248

[8] Giuliano AE Connolly JL Edge SB Mittendorf EA Rugo HS Solin LJ. Breast cancer-major changes in the American Joint Committee on Cancer eighth edition cancer staging manual. CA Cancer J Clin. (2017) 67:290–303. 10.3322/caac.21393 28294295

[9] Thomas A Routh ED Pullikuth A Jin G Su J Chou JW. Tumor mutational burden is a determinant of immune-mediated survival in breast cancer. OncoImmunology. (2018) 7:e1490854. 10.1080/2162402X.2018.1490854 30386679 PMC6207420

[10] Li H Gao C Liu L Zhuang J Yang J Liu C Zhou C Feng F Sun C. 7-lncRNA assessment model for monitoring and prognosis of breast cancer patients: based on cox regression and co-expression analysis. Front Oncol. (2019) 9:1348. 10.3389/fonc.2019.01348 31850229 PMC6901675

[11] Wang S Zhang Q Yu C Cao Y Zuo Y Yang L. Immune cell infiltration-based signature for prognosis and immunogenomic analysis in breast cancer. Brief Bioinform. (2020) 22(2):2020–2031. 10.1093/bib/bbaa026 32141494

[12] Nakaoka T Saito Y Saito H. Aberrant DNA methylation as a biomarker and a therapeutic target of cholangiocarcinoma. Int J Mol Sci. (2017) 18:1111. 10.20944/preprints201705.0127.v1 28545228 PMC5485935

[13] Du T Liu B Wang Z Wan X Wu Y. CpG methylation signature predicts prognosis in breast cancer. Breast Cancer Res Treat. (2019) 178:565–72. 10.1007/s10549-019-05417-3 31520283

[14] Álvarez-Errico D Vento-Tormo R Sieweke M Ballestar E. Epigenetic control of myeloid cell differentiation, identity and function. Nat Rev Immunol. (2015) 15:7–17. 10.1038/nri3777 25534619

[15] Morales-Nebreda L McLafferty FS Singer BD. DNA methylation as a transcriptional regulator of the immune system. Transl Res. (2019) 204:1–18. 10.1016/j.trsl.2018.08.001 30170004 PMC6331288

[16] Tian Y Morris TJ Webster AP Yang Z Beck S Feber A. ChAMP: updated methylation analysis pipeline for Illumina BeadChips. Bioinformatics. (2017) 33:3982–4. 10.1093/bioinformatics/btx513 28961746 PMC5860089

[17] Lê S Josse J Husson F. FactoMineR: an R package for multivariate analysis. J Stat Softw. (2008) 25:1–18. 10.18637/jss.v025.i01

[18] Hänzelmann S Castelo R Guinney J. GSVA: gene set variation analysis for microarray and RNA-Seq data. BMC Bioinformatics. (2013) 14:7. 10.1186/1471-2105-14-7 23323831 PMC3618321

[19] Charoentong P Finotello F Angelova M Mayer C Efremova M Rieder D. Pan-cancer immunogenomic analyses reveal genotype-immunophenotype relationships and predictors of response to checkpoint blockade. Cell Rep. (2017) 18:248–62. 10.1016/j.celrep.2016.12.019 28052254

[20] Zhang C Li Z Qi F Hu X Luo J. Exploration of the relationships between tumor mutation burden with immune infiltrates in clear cell renal cell carcinoma. Ann Transl Med. (2019) 7:648–8. 10.21037/atm.2019.10.84 31930049 PMC6944593

[21] Ritchie ME Phipson B Wu D Hu Y Law CW Shi W. Limma powers differential expression analyses for RNA-sequencing and microarray studies. Nucleic Acids Res. (2015) 43:e47. 10.1093/nar/gkv007 25605792 PMC4402510

[22] Yu G Wang LG Han Y He QY. ClusterProfiler: an R package for comparing biological themes among gene clusters. OMICS J Integrative Biol. (2012) 16:284–7. 10.1089/omi.2011.0118 22455463 PMC3339379

[23] Andersen PK Gill RD. Cox's Regression Model for counting processes: a large sample study. Ann Stat. (2007) 10:1100–20. 10.1214/aos/1176345976

[24] Therneau TM Grambsch PM. Modeling Survival Data: Extending the Cox Model. New York, NY: Springer New York (2000).

[25] Heagerty PJ Lumley T Pepe MS. Time-dependent ROC curves for censored survival data and a diagnostic marker. Biometrics. (2000) 56:337–44. 10.1111/j.0006-341X.2000.00337.x 10877287

[26] Uno H Tian L Cai T Kohane IS Wei LJ. A unified inference procedure for a class of measures to assess improvement in risk prediction systems with survival data. Stat Med. (2013) 32:2430–42. 10.1002/sim.5647 23037800 PMC3734387

[27] Farlik M Halbritter F Müller F Choudry FA Ebert P Klughammer J. DNA methylation dynamics of human hematopoietic stem cell differentiation. Cell Stem Cell. (2016) 19:808–22. 10.1016/j.stem.2016.10.019 27867036 PMC5145815

[28] Binnewies M Roberts EW Kersten K Chan V Fearon DF Merad M. Understanding the tumor immune microenvironment (TIME) for effective therapy. Nat Med. (2018) 24:541–50. 10.1038/s41591-018-0014-x 29686425 PMC5998822

[29] Yoshihara K Shahmoradgoli M Martínez E Vegesna R Kim H Torres-Garcia W. Inferring tumour purity and stromal and immune cell admixture from expression data. Nat Commun. (2013) 4:1–11. 10.1038/ncomms3612 24113773 PMC3826632

[30] Newman AM Steen CB Liu CL Gentles AJ Chaudhuri AA Scherer F. Determining cell type abundance and expression from bulk tissues with digital cytometry. Nat Biotechnol. (2019) 37:773–82. 10.1038/s41587-019-0114-2 31061481 PMC6610714

[31] Li T Fu J Zeng Z Cohen D Li J Chen Q. TIMER2.0 for analysis of tumor-infiltrating immune cells. Nucleic Acids Res. (2020) 48:W509–14. 10.1093/nar/gkaa407 32442275 PMC7319575

[32] Zardavas D Irrthum A Swanton C Piccart M. Clinical management of breast cancer heterogeneity. Nat Rev Clin Oncol. (2015) 12:381–94. 10.1038/nrclinonc.2015.73 25895611

[33] Gannon LM Cotter MB Quinn CM. The classification of invasive carcinoma of the breast. Expert Rev Anticancer Ther. (2013) 13:941–54. 10.1586/14737140.2013.820577 23984896

[34] Liu F Lang R Zhao J Zhang X Pringle GA Fan Y. CD8+ cytotoxic T cell and FOXP3+ regulatory T cell infiltration in relation to breast cancer survival and molecular subtypes. Breast Cancer Res Treat. (2011) 130:645–55. 10.1007/s10549-011-1647-3 21717105

[35] Peng GL Li L Guo YW Yu P Yin XJ Wang S. CD8+ cytotoxic and FoxP3+ regulatory T lymphocytes serve as prognostic factors in breast cancer. Am J Transl Res. (2019) 11:5039–53. 31497220 PMC6731430

[36] Gotwals P Cameron S Cipolletta D Cremasco V Crystal A Hewes B. Prospects for combining targeted and conventional cancer therapy with immunotherapy. Nat Rev Cancer. (2017) 17:286–301. 10.1038/nrc.2017.17 28338065

[37] Kim JE Patel MA Mangraviti A Kim ES Theodros D Velarde E. Combination therapy with anti-PD-1, anti-TIM-3, and focal radiation results in regression of murine gliomas. Clin Cancer Res. (2017) 23:124–36. 10.1158/1078-0432.CCR-15-1535 27358487 PMC5735836

[38] Lee L Matulonis U. Immunotherapy and radiation combinatorial trials in gynecologic cancer: a potential synergy? Gynecol Oncol. (2019) 154:236–45. 10.1016/j.ygyno.2019.03.255 30995960

[39] Germain D. Estrogen carcinogenesis in breast cancer. Endocrinol Metab Clin North Am. (2011) 40:473–84. 10.1016/j.ecl.2011.05.009 21889715

[40] Kovats S. Estrogen receptors regulate innate immune cells and signaling pathways. Cell Immunol. (2015) 294:63–9. 10.1016/j.cellimm.2015.01.018 25682174 PMC4380804

[41] Svoronos N Perales-Puchalt A Allegrezza MJ Rutkowski MR Payne KK Tesone AJ. Tumor cell–independent estrogen signaling drives disease progression through mobilization of myeloid-derived suppressor cells. Cancer Discov. (2017) 7:72–85. 10.1158/2159-8290.CD-16-0502 27694385 PMC5222699

[42] Segovia-Mendoza M Morales-Montor J. Immune tumor microenvironment in breast cancer and the participation of estrogens and its receptors into cancer physiopathology. Front Immunol. (2019) 10:348. 10.3389/fimmu.2019.00348 30881360 PMC6407672

[43] Fukumura D Kloepper J Amoozgar Z Duda DG Jain RK. Enhancing cancer immunotherapy using antiangiogenics: opportunities and challenges. Nat Rev Clin Oncol. (2018) 15:325–40. 10.1038/nrclinonc.2018.29 29508855 PMC5921900

[44] Soria JC Marabelle A Brahmer JR Gettinger S. Immune checkpoint modulation for non-small cell lung cancer. Clin Cancer Res. (2015) 21:2256–62. 10.1158/1078-0432.CCR-14-2959 25979932

[45] Xu F Jin T Zhu Y Dai C. Immune checkpoint therapy in liver cancer. J Exp Clin Cancer Res. (2018) 37:110. 10.1186/s13046-018-0777-4 29843754 PMC5975687

[46] Dempke WCM Fenchel K Uciechowski P Dale SP. Second- and third-generation drugs for immuno-oncology treatment—The more the better? Euro J Cancer. (2017) 74:55–72. 10.1016/j.ejca.2017.01.001 28335888

[47] Sigalotti L Fratta E Coral S Maio M. Epigenetic drugs as immunomodulators for combination therapies in solid tumors. Pharmacol Ther. (2014) 142:339–50. 10.1016/j.pharmthera.2013.12.015 24384533

[48] Mavaddat N Pharoah PDP Michailidou K Tyrer J Brook MN Bolla MK. Prediction of breast cancer risk based on profiling with common genetic variants. J Natl Cancer Inst. (2015) 107(5):djv036. 10.1093/jnci/djv036 25855707 PMC4754625

[49] Lai J Chen B Zhang G Wang Y Mok H Wen L. Identification of a novel microRNA recurrence-related signature and risk stratification system in breast cancer. Aging. (2019) 11:7525–36. 10.18632/aging.102268 31548433 PMC6781975

[50] Muntasell A Rojo F Servitja S Rubio-Perez C Cabo M Tamborero D. NK cell infiltrates and HLA class I expression in primary HER2 þ breast cancer predict and uncouple pathological response and disease-free survival. Clin Cancer Res. (2019) 25:1535–45. 10.1158/1078-0432.CCR-18-2365 30523021

[51] Szyf M. DNA methylation signatures for breast cancer classification and prognosis. Genome Med. (2012) 4:26. 10.1186/gm325 22494847 PMC3446276

[52] Tao C Luo R Song J Zhang W Ran L. A seven-DNA methylation signature as a novel prognostic biomarker in breast cancer. J Cell Biochem. (2020) 121:2385–93. 10.1002/jcb.29461 31646666

