# Supplementary material for: Role of Immune Cell-Specific Hypermethylation Signatures in Classification and Risk Stratification of Breast Cancer
Source: Front Med (Lausanne). 2021 Aug 26;8:674338. doi: 10.3389/fmed.2021.674338 (PMC8426625; doi:10.3389/fmed.2021.674338)
Supplement: Supplementary file 1 [file Table_1.docx]

**Supplementary Table 1**

#### code for calculating the enrichment scores of 26 immune cell types with the method ssGSEA

library(tidyverse)

## 1.load the list of immune cell markers

cellMarker <- data.table::fread("cellMarker.csv",data.table = F)

colnames(cellMarker)[2] <- "celltype"

cell.list <- split(cellMarker,cellMarker$celltype)

cellMarker <- lapply(cell.list, function(x){

dd = x$Metagene

unique(dd)

})

## load the mRNA expression matrx

load('mRNA.matrix.fpkm.RData')

expr <- as.matrix(mRNA.matrix.fpkm)

expr <- log2(expr + 0.01)

## ssGSEA calculation

library(GSVA)

gsva_data <- gsva(expr,cellMarker, method = "ssgsea")

gsva.score <- as.data.frame(t(gsva_data)) %>%

rownames_to_column('sample')

save(gsva.score, file = 'gsva.score.rda')
