# Supplementary material for: Role of Immune Cell-Specific Hypermethylation Signatures in Classification and Risk Stratification of Breast Cancer
Source: Front Med (Lausanne). 2021 Aug 26;8:674338. doi: 10.3389/fmed.2021.674338 (PMC8426625; doi:10.3389/fmed.2021.674338)
Supplement: Supplementary file 2 [file Table_2.docx]

**Supplementary Table 2**

1. **The equation for constructing multiple Cox model**

The most commonly used models for survival data are those that model the transition rate from state to state, i.e. They are Poisson regression (3.1), the Cox or proportional hazards model (3.2) and the Aalen additive regression model (3.3), of which the Cox model is far and away the most popular. As seen in the equations they are closely related.

| *λ*(*t*) | = *ebeta*0 +*β*1 *x*1 +*β*2 *x*2 +*...* | (3.1) |
| --- | --- | --- |
| *λ*(*t*) | = *eβ*0 (*t*)+*β*1 *x*1 +*β*2 *x*2 +*...* |  |
|  | = *λ*0(*t*)*eβ*1 *x*1 +*β*2 *x*2 +*...* | (3.2) |

*λ*(*t*) = *β*_0_(*t*) + *β*_1_(*t*)*x*_1_ + *β*_2_(*t*)*x*_2_ + *. . .* (3.3)

(Note: these formulas come from the studies of Andersen PK et al.^1^ and Therneau TM et al.^2^, and are applied in the R package “survival”)

1. **The equation for estimating ROC curves**

We outline an approach for the estimation of *ROC* curves with a time-dependent disease variable, or more generally a failure time. This approach is based on direct use of Bayes’ theorem and the Kaplan-Meier estimator.

2.1 Definitions

Let *Ti* denote failure time and *Xi* the covariate value for subject *i*. Let *Ci* denote the censoring time, *Zi* = min (*Ti*, *Ci*) the follow-up time, and *δi* a censoring indicator with *δi* = 1 if *Ti* ≤ *Ci* and *δi* = 0 if *Ti > Ci*. We use the counting process *Di(t)* = 1 if *Ti* ≤ *t* and *Di(t)* = 0 if *Ti* > *t* to denote failure (disease) status at any time *t* with *Di(t)* = 1 indicating that subject *i* has had an event prior to time *t*.

Recall that ROC curves display the relationship between a covariate *Xi*, and a binary disease variable *Di* by plotting estimates of the sensitivity, *P(X > c* | *D* = 1*)*, and one minus the specificity, 1 - *P*(*X* ≤ c | *D* = 0) for all possible values *c*. When disease status is time-dependent, consider sensitivity and specificity as time-dependent functions and define them as


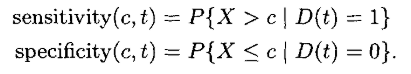


Using these definitions, we can define the corresponding ROC curve for any time *t*, ROC*(t)*.

2.2 Using the Kaplan-Meier Estimator

We can use Bayes’ theorem to rewrite the sensitivity and the specificity as


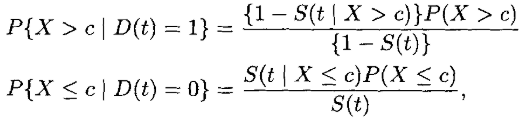


where *S(t)* is the survival function *S(t)* = *P*(*T* > *t*) and *S*(*t* | *X* > *c*) is the conditional survival function for the subset defined by *X* > *c*. A widely used nonparametric estimate of *S(t)* is given by

Kaplan and Meier (1958). Define *Tn* to be the unique values of *Zi* for observed events, *δi* = 1. The Kaplan-Meier (KM) estimator is defined as


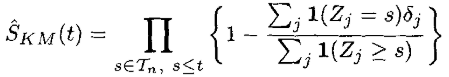


The KM estimator uses all of the information in the data, including censored observations, to estimate the survival function. A simple estimator for sensitivity and specificity at time *t* is then given by combining the KM estimator and the empirical distribution function of the marker covariate, *X*, as


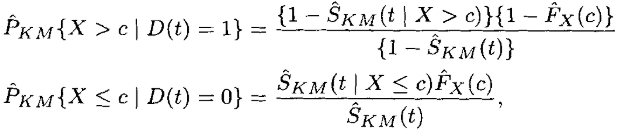


Where *x(c)* = Σ 1 (*Xi* ≤ *c*)/*n.*

(Note: these formulas come from the study of Heagerty PJ et al.^3^ and is applied in the R package “survivalROC”)

References:

1. Andersen PK, Gill RD. Cox’s Regression Model for Counting Processes: A Large Sample Study. *The Annals of Statistics*. 2007;10(4):1100-1120. doi:10.1214/aos/1176345976

2. Therneau TM, Grambsch PM. *Modeling Survival Data: Extending the Cox Model*. New York, NY: Springer New York; 2000. doi:10.1007/978-1-4757-3294-8

3. Heagerty PJ, Lumley T, Pepe MS. Time-dependent ROC curves for censored survival data and a diagnostic marker. *Biometrics*. 2000;56(2):337-344. doi:10.1111/j.0006-341X.2000.00337.x
