# Supplementary material for: Role of Immune Cell-Specific Hypermethylation Signatures in Classification and Risk Stratification of Breast Cancer
Source: Front Med (Lausanne). 2021 Aug 26;8:674338. doi: 10.3389/fmed.2021.674338 (PMC8426625; doi:10.3389/fmed.2021.674338)
Supplement: Supplementary file 8 [file Data_Sheet_1.pdf]

Supplementary Figures

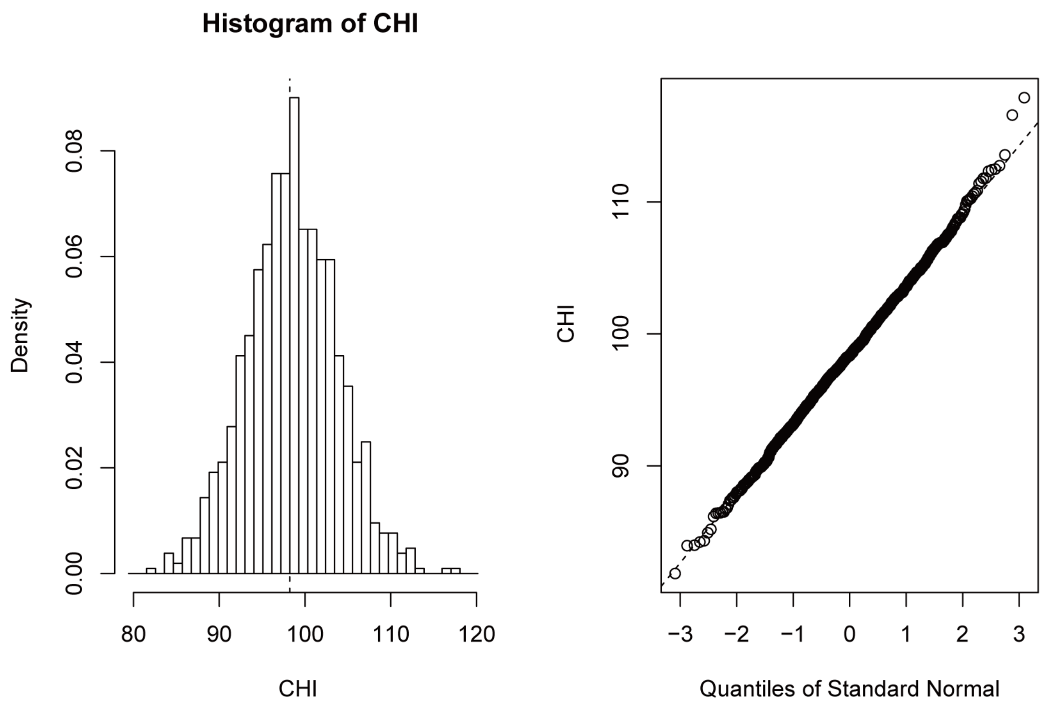

Supplementary Figure 1: Histogram of 1000 CHIs derived from the resampling datasets (A), and normal quantile plot shows that the distribution of CHIs is normally distributed (B).

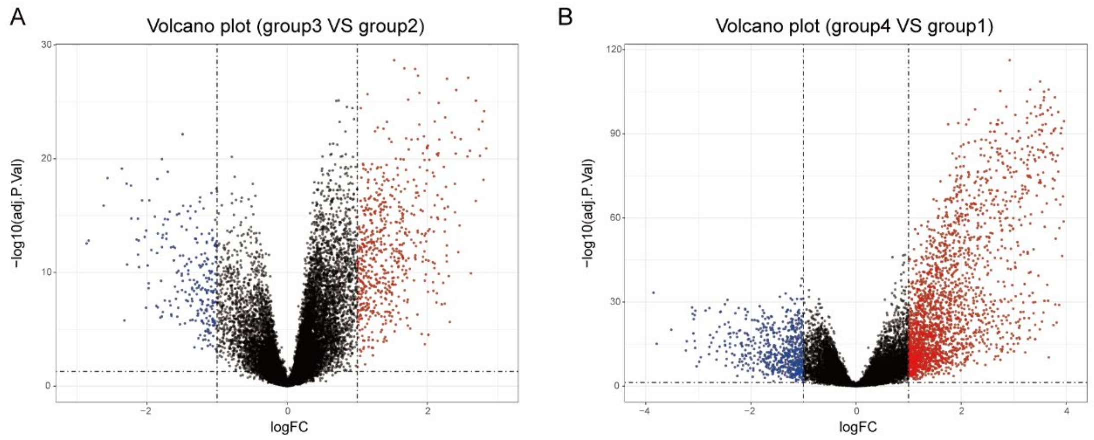

Supplementary Figure 2: Volcano plots of differential expressed genes between groups 3 and 2 (A), and between groups 4 and 1 (B).

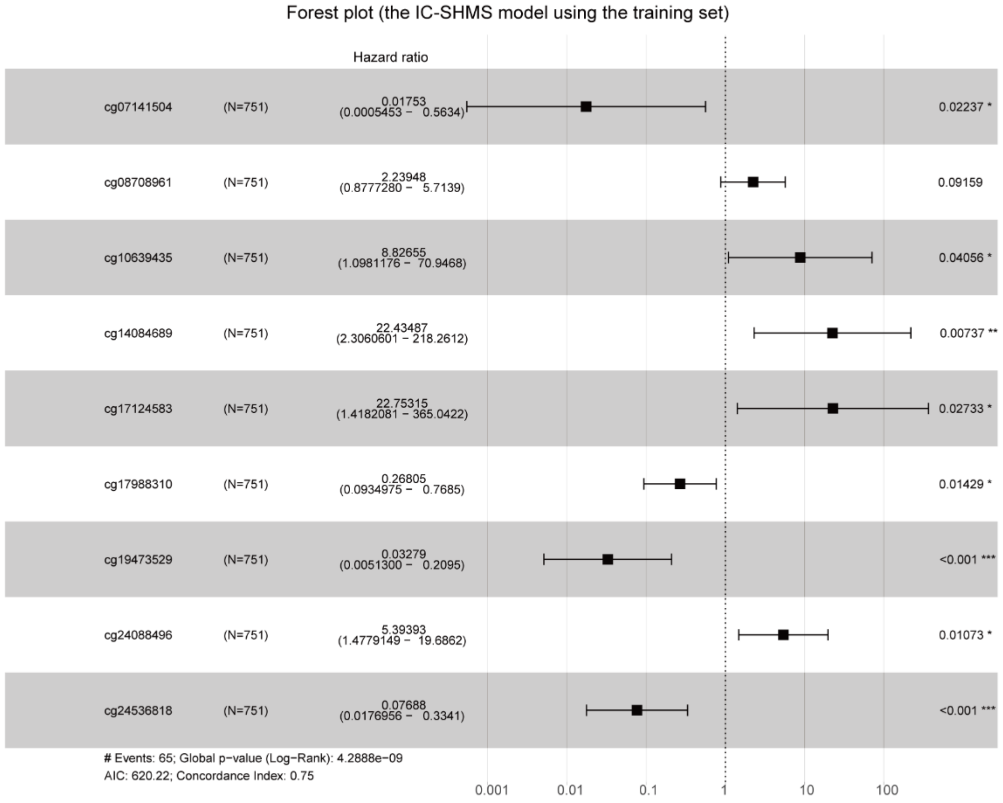

Supplementary Figure 3: Forest plot showing the hazard ratios of the nine risk features included in the IC-SHMS model derived from the training set.

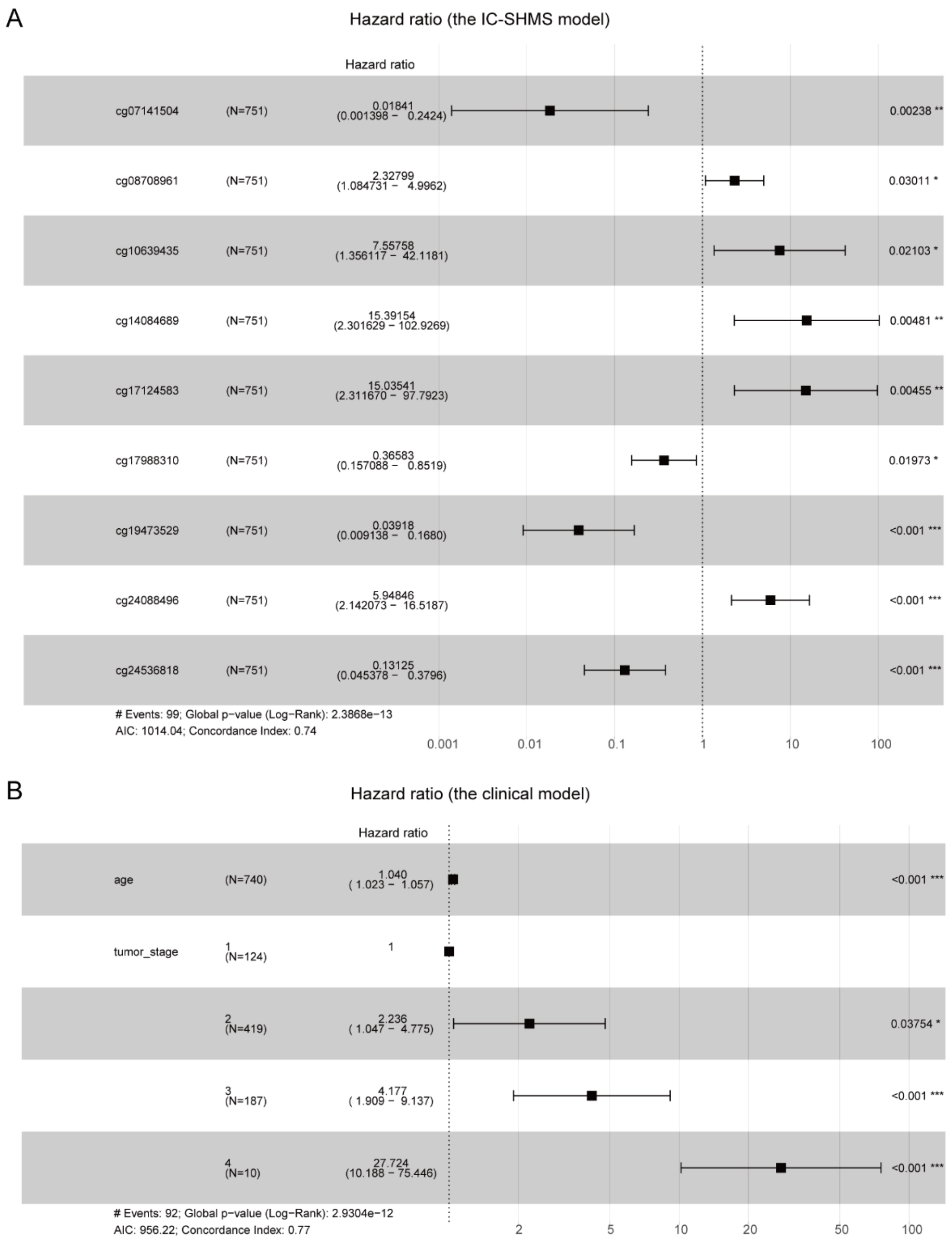

Supplementary Figure 4: Forest plots showing the hazard ratios of the nine risk features included in the IC-SHMS model (A), and the hazard ratios of the two risk features included in the clinical model (B).

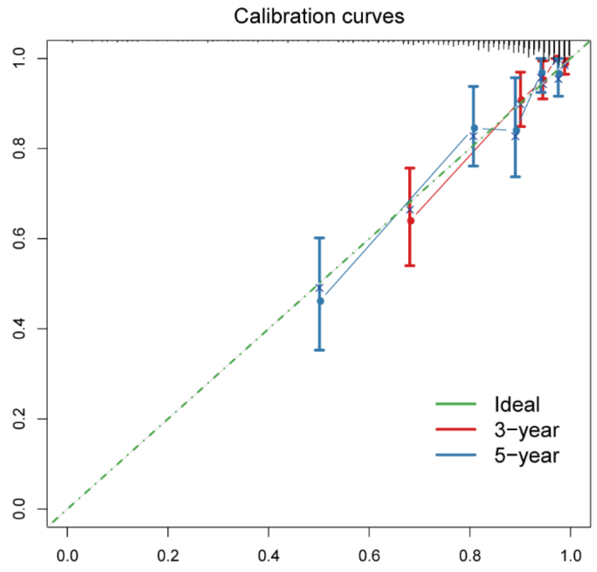

Supplementary Figure 5: Calibration curves showing that the combined model had a good performance in survival prediction accuracy of the 3- and 5-year OS compared with the ideal model.
